# A systematic review of head-up tilt to improve consciousness in people with a prolonged disorder of consciousness

**DOI:** 10.1177/0269215520946696

**Published:** 2020-07-30

**Authors:** Harriet Ng, Andrew King

**Affiliations:** 1 Centre for Sport, Exercise and Life Sciences (CSELS), Coventry University, Coventry, Warwickshire, UK; 2 Coventry University, Coventry, Warwickshire, UK

**Keywords:** Traumatic brain injury, prolonged disorders of consciousness, acquired brain injury, physiotherapy, rehabilitation

## Abstract

**Objective:**

This systematic review analysed the evidence for the effect of head-up tilt (passive-standing) on consciousness among persons in prolonged disorders of consciousness.

**Data sources:**

Articles were identified through primary database searching (Medline, CINAHL, AMED, The Cochrane Library) and post-citation searching (Scopus).

**Review methods:**

This review followed the PRISMA statement. The search strategy was created to find articles that combined any conceivable passive standing device, any measure of consciousness and disorders of consciousness of any origin. Inclusion criteria were any papers that evaluated the use of head-up tilt in adults in defined disorders of consciousness. Exclusion criteria included active stand studies, paediatric studies and animal studies.

The search was completed independently by two researchers. Data collection and risk of bias assessment was completed using the Downs and Black tool.

**Results:**

6867 titles were retrieved (last search completed 21/6/20). Ten papers met the inclusion criteria: five examined the effects of a single head-up tilt treatment, and five the effects of head-up tilt regimes. Eighty-seven participants were randomised in three randomised controlled trials. In the remaining preliminary studies or case series, 233 participants were analysed. Quality was low, with only two high-quality studies available. Four studies were suitable for effect size analysis, where medium to large effect sizes were found. The two high-quality studies found head-up tilt had a large effect on consciousness.

**Conclusion:**

Overall there is some evidence that repeated passive standing on a tilt-table can improve consciousness, but the relevant studies provoke further questions.

## Introduction

A consequence of severe brain injuries is a prolonged disorder of consciousness.^1^ The primary aim of rehabilitation for people in disorders of consciousness is to increase their levels of alertness. However, at present there is a scarcity of evidence-based treatment options for persons in disorders of consciousness.^2^

A tilt-table is a motorised bed with straps and a foot-plate that can be used to elevate people who are unable to stand on their own.^3^ Recently new equipment has been developed to allow therapists to incorporate passive stepping while standing a person on a tilt-table, using a motorised footplate (Erigo).^4^ Previous systematic reviews have assessed the ability of passive standing to improve common treatment objectives, such as maintaining soft tissue length in the lower limb, lung function, circulation and gastrointestinal tract.^5–7^ Tilt-table treatment is usual practice, and therapists have good access to such equipment. In the UK, 66% of physiotherapists had access to a tilt-table.^8^ In Australia, Chang et al.^9^ found 67.4% of respondents completed acute head-up tilt using the tilt-table.

Head-up tilt is commonly also used in rehabilitation with the aim of improving consciousness. Several authors have championed head-up tilt’s ability to affect consciousness, but no systematic review has evaluated the strength of this literature. Two surveys in Australia and the United Kingdom found raising consciousness to be one of the five most-cited reasons for the use tilt-table training.^8,9^ However, while the treatment goal to increase levels of consciousness is commonplace, the evidence underpinning this practice is limited.

## Methods

### Search strategy

Two investigators independently performed the search (last search completed 21/6/20). The search and reporting followed the PRISMA statement for systematic reviews. Funding to undertake this systematic review was provided by Health Education England and the National Institute of Health Research. This systematic review was registered in PROSPERO (registration number: CRD42018084069).

Four databases were searched: Medline, CINAHL, AMED and The Cochrane Library. The search was completed using the main key words pertaining to the population, intervention and outcome. In addition to this, key words from the randomised controlled trials were used to improve the search strategy. The five main causes of a prolonged disorders of conscious were used to inform the population: this included a vascular event, traumatic brain injury, hypoxia, infection and toxic/metabolic aetiologies. All common variations of tilt-table devices and standing frames were searched for. For results related to the main outcome, the search included all measurements of consciousness including neurobehavioural tools.

No limit was set on language, publication date or study quality. Hence preliminary studies and case series were included. The reference lists of the selected articles were screened along with the key authors’ previous works to ensure all relevant articles were retrieved.

The articles retrieved were screened by title and then abstract independently by the two investigators. If this did not provide sufficient information the full text was reviewed. The two reviewers came to a consensus through discussion. Articles were included if they met the ‘P.I.C.O’ inclusion criteria.^10^ The study population was adults (>18 years old) of either gender with a definitive diagnosis of coma, Vegetative State ‘VS’ or Minimally Conscious State ‘MCS’.^11^ The primary outcome of interest was change in consciousness as measured by neurobehavioural assessment, or physiological change linked to consciousness. Appropriate comparison was with traditional physiotherapy, physical therapy treatments or differing head-up tilt devices. Articles were included if they evaluated consciousness using an appropriate outcome measure on this population undergoing head-up tilt treatment. There was no limit on study setting, quality, date or language. Paediatric studies were excluded due to the differing recovery of an immature brain. Post-citation searching of included studies was then performed using Scopus. This also involved searching the main authors’ published literature and any document that had cited the main author.

Data collection and methodological quality of the selected articles was evaluated independently by the two examiners using the Downs and Black checklist.^12^ The maximum score on this scale is 28 points.^12^ The following categories were used to classify study quality; poor (14), fair (15–19), good (20–25) and excellent (26–28).^13^ The Downs and Black checklist has been shown to have high internal consistency and good test-retest and inter-rater reliability.^12^ For all articles each examiner completed data extraction which included information on the participants, methodology, type of tilting device, outcomes, main findings, risk of bias and data analysis. Any differences in scoring between reviewers was resolved through re-referring to the articles during consensus meetings.

None of the studies included were sufficiently homogenous to allow meta-analysis or subgroup analysis. Where possible the between-groups and within-groups effect sizes were analysed using Cohen’s *d* statistic.^14^ Where there was no control group, the pre- and post-treatment scores were used to calculate the mean difference.

## Results

**Figure 1.**
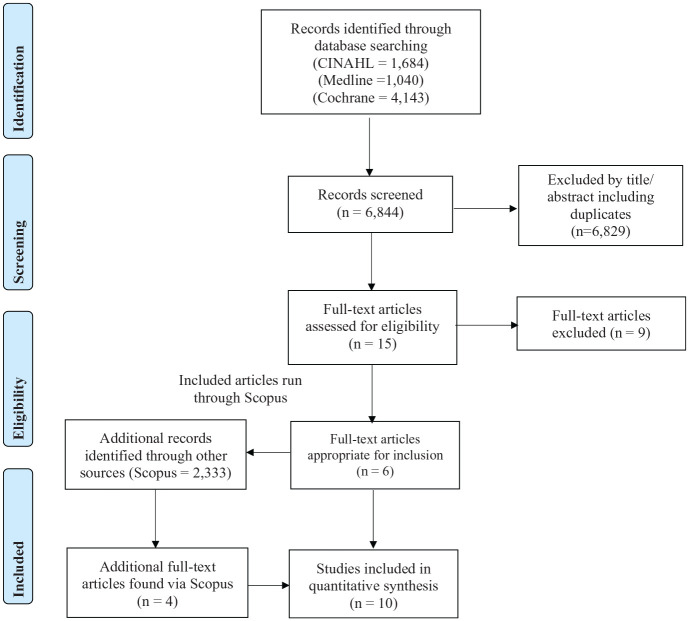
PRISMA flow diagram.

6867 titles were retrieved from the databases and filtered in turn using title and abstract. Fifteen full text articles were reviewed, and six met the inclusion criteria. Subsequent post-citation searching of the articles found a further four articles.^15^ The PRISMA diagram depicts the search strategy (Figure 1). No systematic reviews or grey literature met the inclusion criteria. Of the 10 selected articles, five studied the effects on consciousness of a singular head-up tilt treatment (Table 1) and five articles assessed consciousness after up-to-four weeks of a head-up tilt regime (Table 2).

**Table 1.** Characteristics of studies of head-up tilt single session.

Authors	Study type, location, setting	Outcome measures for consciousness	Enrolled sample; number, average age, male/female and consciousness state	Intervention
Elliott et al.^18^	Case series UK Not stated	Wessex Head Injury Matrix	**Number = 12** Ages 19 to 71 years 8 Males, 4 Females Vegetative State (*n* = 5) Minimally Conscious State (*n* = 7)	**Device:** Tilt-table **Maximum elevation:** 85° **Consciousness assessment:** 1. Supine 2. Head-up tilt
Greco et al.^17^	Case series Italy Brain Injury Unit	Electroencephalogram Power spectral analysis Symmetry index	**Number = 3** Ages 56 to 73 years 1 Male, 2 Females Minimally Conscious State (*n* = 3)	**Device:** Erigo **Maximum elevation:** 60° **Consciousness assessment:** During head-up tilt
Luther et al.^4^	Randomised crossover pilot trial Germany Neuro-rehabilitation hospital	Coma Recovery Scale-Revised	**Number = 9** Ages 20 to 51 years 5 Males, 4 Females Vegetative State (*n* = 3) Minimally Conscious State (*n* = 6)	**Device:** 1. Tilt-table 2. Erigo **Maximum elevation:** 70° **Consciousness assessment:** Time not stated.
Riberholt et al.^21^	Case series Denmark Traumatic Brain Injury Unit	% time period with eyes open	**Number = 16** Ages 18 to 74 years 10 Males, 6 Females Vegetative State (*n* = 3) Minimally Conscious State (*n* = 11) Aware (*n* = 2)	**Device:** Tilt-table **Maximum elevation:** 80° **Consciousness assessment**: 1. 30 minutes before head-up tilt 2. During head-up tilt
Wilson et al.^19^	Case series United Kingdom Not stated	Wessex Head Injury Matrix	**Number = 16** Ages 27 to 70 years 10 Males, 6 Females Vegetative State (*n* = 8) Minimally Conscious State (*n* = 8)	**Device:** Tilt-table **Maximum elevation:** 90° **Consciousness assessment**: 1. Supine 2. Sitting (without head support) 3. During head-up tilt

**Table 2.** Characteristics of studies of head-up tilt regimes.

Author	Study type, location, setting	Outcome measures for consciousness	Enrolled sample; number, average age, male/female and consciousness state	Intervention
Bartolo et al.^16^	Prospective observational study Italy Neurological Intensive Care Units (14 units)	Glasgow Coma Scale Disability Rating Scale Rancho Los Amigos Levels of Cognitive Functioning Scale Early Rehabilitation Barthel Index Glasgow Outcome scale	**Number = 102** Ages 45.6 to 69.7 years 60 Males, 42 Females Glasgow Coma Scale at baseline 6.5 (5.7–7.3)	**Device:** Tilt-table **Maximum elevation:** ⩾40° **Allocated to** • Early mobilisation group – sitting on the bed or standing using a tilt-table to ⩾40° • Non-mobilisation group **Consciousness assessment:** Baseline, 1st evaluation, 2nd evaluation, 3rd evaluation, 4th evaluation and Intensive Care Unit discharge. **Number of sessions:** Not stated.
Frazzitta et al.^22^	Single-blind randomised clinical trial Italy Intensive Care Unit	Coma Recovery Scale-Revised Disability Rating Scale Rancho Los Amigos Levels of Cognitive Functioning Scale	**Number = 31** Ages 22 to 82 years 20 Males, 11 Females Vegetative State (*n* = 31)	**Device:** Erigo **Maximum elevation**: 60° **Randomisation to** • Head-up tilt plus traditional physiotherapy • Conventional in-bed physiotherapy **Consciousness assessment:** Admission, Intensive Care Unit discharge and Neurorehabilitation discharge. **Number of sessions:** 15
Krewer et al.^24^	Single blind Randomised Controlled Trial Germany Rehabilitation unit	Coma Recovery Scale-Revised	**Number = 44** Ages 23 to 74 years 26 Males, 18 Females Vegetative State (*n* = 14) Minimally Conscious State (*n* = 30)	**Device(s):** Tilt-table or Erigo **Maximum elevation:** 70° **Randomisation to** • Tilt-table regime • Erigo regime **Consciousness assessment:** Baseline, week 3 and week 6. **Number of sessions:** 20 sessions
Taveggia et al.^20^	Randomised controlled trial Italy Neuro-rehabilitation hospital	Coma Recovery Scale-Revised Rancho Los Amigos Levels of Cognitive Functioning Scale	**Number = 8** Ages 47 to 79 years 4 Males, 4 Females Coma Recovery Scale-Revised not recorded prior to treatment.	**Device(s):** Tilt-table or Erigo **Maximum elevation: 65**° **Randomisation to** • Study group A – Erigo • Control group B – Tilt-table Then each group repeated the other treatment method. **Consciousness assessment:** Before and after treatment on each device. **Number of sessions:** 24
Toccolini et al.^23^	Prospective cohort study Brazil Intensive care unit	Glasgow Coma Scale	**Number = 23** Ages 42.5 to 79.5 years 15 Males, 8 Females Glasgow Coma Scale not recorded prior to treatment.	**Device:** Tilt-table **Maximum elevation:** 90° **Consciousness assessment:** 1. Day 1, day 2 and day of discharge and 2. At each inclination (30°, 45°, 60°, 75° and 90°) **Number of sessions:** Varied between patients (daily until discharge from Intensive Care Unit).

The total number of participants included was 264, with 159 males and 105 females. Bartolo et al.^16^ had the largest sample size of 102 and Greco et al.^17^ the smallest with three participants. The majority of studies had 16 participants or fewer.^18–21^ The age range of study participants was from 19 to 82 years. The causes of prolonged disorders of consciousness were highly varied. The most frequently recorded aetiologies were traumatic brain injury (27%), haemorrhagic (21%), non-traumatic brain injury (15%) and ischaemic injury (10%).

Head-up tilt was achieved by a variety of methods. Many researchers elevated their participants incrementally from horizontal (0°) to 60/90° in allocated time intervals. Taveggia et al.^20^ completed a change in tilt every 10 minutes from 30° to 65° and then maintained this elevation for 30-minutes. Greco et al.^17^ raised the tilt-table every five minutes. Riberholt et al.^21^ performed elevation to 30°, 60° and then 80° in 60-second intervals. Many authors took their participants up incrementally but did not state their rate or degree of inclination.^22,23^ Two authors performed elevation when participants adjusted physiologically, not in time intervals. ^4,19^ All articles varied in the intricacies of their elevation methods.

The duration of head-up tilt for single treatments was between 10 and 20 minutes.^17,18,21^ Two studies did not report a specific duration. ^4,19^ For head-up tilt treatment regimes, the duration of inclination varied between 30 minutes^22,23^ and an hour of net therapy time.^24^ Exact intervention timings were not given by Bartolo et al.^16^

The type of device used for elevation was either a tilt-table or Erigo. Standing was achieved via tilt-table in five studies.^18,19,21,23^ Greco et al.^17^ used solely the Erigo with their participants. Three studies compared the effects of the tilt-table and Erigo^20,24^ and one study compared the effects of the Erigo to a control group.^22^

Luther et al.,^4^ Krewer et al.^24^ and Taveggia et al.^20^ compared the effects of tilt table elevation with head-up tilt on the Erigo. Luther et al.^4^ had six participants who had orthostatic hypotension on the tilt table but not the Erigo. Krewer et al.^24^ had on average a significantly longer duration of net therapy time on the Erigo (25 minutes) in comparison to the tilt table (23 minutes). This increase in therapy time was due to the reduction of interruptions, which were less frequent on the Erigo at 15.4% compared to 32.3% on the tilt table. Taveggia et al.^20^ found using the Erigo prevented neurally mediated syncope in haemodynamically unstable patients. In all studies the Erigo produced a reduction in presyncopal symptoms and interruptions to treatment due to orthostatic hypotension.

The methodological quality of the studies was assessed using the Downs and Black Checklist^12^ (Table S1) and given corresponding quality levels via secondary classification (Table S2).^13^

Blinded random allocation to multiple groups was completed in four studies^4,20,22,24^ The remainder of the studies had a single arm.^17–19,21,23^ Assessment bias was present in multiple studies. Only two included articles achieved assessor blinding^22,24^ six studies did not blind their assessors^18,19,21,23^ and one study did not provide sufficient information about assessor blinding.^20^ Bartolo et al.^16^ designed their study as a prospective observational study meaning that all assessors were fully aware of the participants’ treatment.

Six out of the 10 studies used appropriate statistical tests to analyse their data.^16,17,21,23^ Luther et al.^4^ and Riberholt et al.^21^ did not have consciousness as their primary outcome and did not publish full statistical tests for their secondary outcome measure of consciousness.

A valid outcome measure was used to assess consciousness in the majority of the included studies.^18,19,24^ Riberholt et al.^21^ used an unvalidated outcome measure: the proportion of time a participant had their eyes open. Greco et al.^17^ stated that their chosen measure, electroencephalogram activity, correlated with alertness in a previous study in normal controls,^25^ but this finding has yet to be validated for those in prolonged disorders of consciousness.

Four out of five studies investigating single head-up tilt treatment sessions found a positive effect; however, these four studies did not have a control group comparison so improvement due to natural recovery cannot be ruled out (Table 3).^17–19,21^ Elliott et al.^18^ reported a relatively large treatment effect when using a tilt-table. Wilson et al.^19^ compared elevation on the tilt-table to sitting, with head-up tilt producing a larger effect size compared to sitting. Riberholt et al.^21^ used the duration of time the participants had their eyes open to show increased levels of arousal. On average the group spent almost three times as long with their eyes open during head-up tilt than beforehand. However, Luther et al.^4^ found no change in consciousness between lying and elevation on a tilt-table or Erigo.

**Table 3.** Results for single head-up tilt studies.

Authors	Outcome measures for consciousness	Authors’ results	Secondary analysis (effect size)
Elliott et al.^18^	Wessex Head Injury Matrix	• No change – 3 participants • 0 to 10 point – 4 participants • 11 to 20 point – 3 participants • 21 to 30 point – 1 participant • 31 to 40 point – none recorded • 41 to 50 point – 1 participant • 51 to 62 point – none recorded	Cohen’s *d* = 0.868 Relatively large treatment effect
Greco et al.^17^	Electroencephalogram Power spectral analysis Symmetry index	• Statistical difference in beta band wavelength, from power spectral analysis and symmetry index between brain hemispheres for all patients.	Unable to calculate due to insufficient data.
Luther et al.^4^	Coma Recovery Scale-Revised	• No change – 7 participants • 0 to 5 point – 3 participants • 6 to 10 point – none recorded • 11 to 16 point – none recorded • 17 to 20 point – none recorded • 21 to 23 point – none recorded	Unable to calculate due to insufficient data.
Riberholt et al.^21^	% time period with eyes open	Proportion of time eyes open before the intervention – average 22.1% of the 30 minutes. During treatment proportion of time with eyes – average 66% of 15 minutes.	(Time with eyes open during treatment / time with eyes open before treatment) × 100 = percentage change of time with eyes open (66% / 22.1%) × 100 = 298%
Wilson et al.^19^	Wessex Head Injury Matrix	Lying to standing • Decrease –5 to 0 point – none recorded • No change – 3 participants • 0 to 3 point – 9 participants • 4 to 6 point – 4 participants • 6 to 62 point – none recorded	Lying to sitting; Cohen’s *d* = 0.367 (medium effect size) Lying to standing; Cohen’s *d* = 0.547 (medium effect size)

For the head-up tilt regimes there were broadly positive trends (Table 4), but these were demonstrated with differing devices and protocols. Frazzitta et al.^22^ showed a large treatment effect on levels of consciousness using an Erigo compared to the control group. Krewer et al.^24^ found only a small treatment effect on the Erigo, but a large effect for the traditional tilt-table. Percentage increases in consciousness measures were demonstrated after early mobilisation in intensive care on a tilt-table in two Italian studies.^16,23^

**Table 4.** Results for head-up tilt regimes.

Authors	Outcome measures for consciousness	Authors’ results	Secondary analysis within group (treatment group)	Secondary analysis within group (control group)
Bartolo et al.^16^	Glasgow Coma Scale	• Average change of 1.6 points for the control group • Average change of 3 points for mobilisation group	Mobilisation group (post treatment–pre treatment)/pre treatment × 100 = (10.3–7.3)/7.3 × 100 = 41.09% change	Non-mobilisation group (post treatment–pre treatment)/pre treatment × 100 = (7.3–5.7)/5.7 × 100 = 28.07% change
Frazzitta et al.^22^	Coma Recovery Scale-Revised	• Average 8 point change for control group • Average 19 point change for mobilisation group	Cohen’s *d* = 2.300 (large treatment effect)	Cohen’s *d* = 1.996 (large treatment effect)
Krewer et al.^24^	Coma Recovery Scale-Revised	0 to 6 weeks tilt-table group change • Decrease –10 to 0 point – 1 participant • No change – none recorded • 0 to 5 point – 5 participants • 6 to 10 point – 14 participants • 10 to 15 point – 2 participants • 15 to 23 point – none recorded 0 to 6 weeks Erigo group change • Decrease –10 to 0 point – 2 participants • No change – none recorded • 0 to 5 point – 9 participants • 6 to 10 point – 8 participants • 10 to 15 point – 2 participants 15 to 23 point – 1 participant	Erigo Cohen’s *d* = 0.180 (small treatment effect)	Tilt-table Cohen’s *d* = 1.934 (large treatment effect)
Taveggia et al.^20^	Coma Recovery Scale-Revised	No numerical data given pre and post intervention for consciousness. Authors state there was no change between groups.	Unable to calculate due to insufficient data.	Unable to calculate due to insufficient data.
Toccolini et al.^23^	Glasgow Coma Scale	Average change on GCS score of 1.7	Elevation to 60° (post treatment–GCS pre treatment)/GCS prior to treatment × 100 = (8.1–5.5)/5.5 × 100 = 47.37% change	N/A

For three studies treatment effect and percentage change could not be calculated due to lack of appropriate data. Greco et al.^17^ performed a single head-up tilt study using electroencephalogram power spectral analysis and symmetry index pre- and post-inclination. The data produced by this study did not allow treatment effect to be calculated. For Luther et al.^4^ and Taveggia et al.^20^ there were no data available from changes on the Coma Recovery Scale-Revised to perform statistical analysis.

The included studies used diverse outcome measures, interventions and assessment. The clinical and methodological heterogeneities were too high to permit a meta-analysis. It was not possible to perform a subgroup analysis into this heterogeneity due to the insufficient numbers of studies and their data being inappropriate for pooling.

## Discussion

The prescription of tilt-table therapies to increase consciousness and treat other outcomes for persons in prolonged disorders of consciousness is commonplace. However, this systematic review has revealed insufficient high-quality evidence to support this practice. In particular, the findings from the case series studies should be reviewed with caution. Many studies had particular methodological flaws, for example, lack of blinded assessment and inappropriate outcome measure selection. There were diverse treatment protocols for all regimes; this included the time taken to maximum elevation, degrees of inclination and total head-up time. Two out of the three randomised controlled trials included head-up tilt in both arms of their study, preventing true control group comparison.^20,24^ Overall, it is not possible to draw definitive conclusions from the quality of this evidence. Further research is required in this area to gain a better understanding of the effectiveness of this intervention in the prolonged disorders of consciousness population.

This systematic review is the first to assess head-up tilt to enhance consciousness in a prolonged disorder of consciousness population. The strengths of this review are the rigorous literature search and reporting using the PRISMA statement.^26^ The search was refined by the key words used in each of the randomised controlled trials and post-citation searching. Full statistical analysis of the studies was not possible, but a thorough narrative analysis has been achieved.

The main limitation of this review was the complexity of the search strategy. In an attempt to find all relevant articles, there was no limit placed on language or creation date. Broad terminology for head injury and standing devices were selected to reflect clinical practice. Despite this, the inconsistency of terminology used complicated the search. These factors increased the number of citations which were time-consuming to review. The narrowing down of appropriate articles was mainly achieved by the small number of studies that used consciousness as an outcome measure. Despite this extensive search, half of studies were found by post-citation searching, which highlights the complexity of searching for articles in this subject area.

There is a distinction between the conclusions that can be drawn about the effect of a single head-up tilt treatment and a cumulative effect of a regime. The single treatments were of lower methodological quality. Choice of outcome measures was inappropriate, and assessors were not blinded. There is insufficient evidence to support improvement in consciousness on a single treatment with head-up tilt.

The tilt-table regime studies were of higher quality, three out of five being randomised controlled trials. Nevertheless, the randomised controlled trials encountered other difficulties which limited their ability to define the effectiveness of head-up tilt treatments. One of these complications was creating two evenly matched groups for comparison. The diversity of persons in prolonged disorders of consciousness causes difficulty in creating two homogenous groups for comparison. Despite their best efforts, Frazzitta et al.^22^ had a control group with a higher average age and a larger number of participants post-haemorrhage. Krewer et al.^24^ also encountered this difficulty, having 10 participants with a potentially worse prognosis in their tilt-table group. This lack of a homogenous study population has reduced authors’ ability to attribute the between-group differences to the research intervention.

Rehabilitative care is complex, particularly when treating persons in prolonged disorders of consciousness. When research is conducted in a rehabilitation setting, regular therapies continue from numerous health professionals. As each participant will receive a unique set of treatments outside of the research interventions, this makes control group comparisons questionable.^25^ Three out of five head-up tilt regimes reported the treatments that occurred alongside the research intervention.^22,24^ However, the duration and the nuances of these treatments were not reported. Since all of these treatments could have affected consciousness, a full report of therapies received would improve transparency.

An important consideration is the type of standing device that could best affect consciousness for the prolonged disorders of consciousness population. Comparisons between the Erigo and the conventional tilt-table were made in three studies.^4,20,24^ These studies compared changes in consciousness and the occurrence of orthostatic hypotension on both devices. All studies found a reduction in discontinuations due to orthostatic hypotension on the Erigo. This is of clinical significance as interruptions to treatment frequently prevent rehabilitation on the tilt table. For patients that are less haemodynamically stable an Erigo may better enable head-up tilt. However, a consistent pattern was not found for consciousness improvement between the tilt-table and Erigo. Luther et al.^4^ and Taveggia et al.^20^ did not find a preferential change in consciousness between the devices. Krewer et al.^24^ found that the tilt-table treatment group had higher recovery of consciousness than the Erigo group. This improved recovery was despite the tilt-table group having a worse prognosis.^12^ This finding could suggest that regular treatments are more important than a longer treatment duration since the tilt-table group had overall a shorter treatment time due to interruptions from orthostatic hypotension. More research is required in this area.

The optimal time to introduce head-up tilt into the rehabilitation regime of persons in prolonged disorders of consciousness has not been explored, but the safety of this treatment has now been investigated. Frazzitta et al.^22^ and Toccolini et al.^23^ achieved the safe introduction of head-up tilt in the acute stages of head injury, with Frazzitta et al.^22^ recruiting participants on the third day after sustaining an acquired brain injury. Toccolini et al.^23^ reported no medical complications on the first elevation to 60°. There were no adverse events reported by Frazzitta et al.^22^ during the stepping verticalisation sessions. These two studies successfully achieved the early and safe introduction of tilt-table elevation.

Measuring consciousness is highly complex.^27^ It is vital that in future investigations of treatment effectiveness that the primary outcome measure is validated. For example, the duration of eye-opening used by Riberholt et al.^21^ is an inadequate measure for assessing active brainstem function, because persons in a vegetative state can have their eyes open but remain unaware of their environment.^28^ Greco et al.^17^ reported an increase in the beta band wavelength during Erigo verticalisation, which they state is associated with human alertness. Although increased activity was seen during head-up tilt, it is still unknown how this correlates with consciousness improvement. A more recent study examining the use of electroencephalogram in the categorisation of vegetative state and minimally conscious state patients found that electroencephalogram can only complement behavioural measures.^29^ Best practice would currently be to use a validated neurobehavioural tool in the assessment of consciousness.

If researchers are to assess consciousness accurately, they need to choose an appropriate measure. Neurobehavioural scales provide a fast and inexpensive measure of consciousness.^30^ A systematic review of neurobehavioural assessment scales concluded that the Wessex Head Injury Matrix and Coma Recovery Scale-Revised have sufficient content validity to assess the four criteria for Vegetative state versus Minimally Conscious State.^30^ These scales are best suited to differentiate a person’s level of consciousness. Overall, Seel et al.^30^ found that the Coma Recovery Scale-Revised was the only assessment scale that could be recommended with minor reservations. Future research should focus on using the Coma Recovery Scale-Revised. The standardised use of the Coma Recovery Scale-Revised in research would allow greater comparison between studies and hence possible meta-analysis.

The finding of Krewer et al.^24^ that the Erigo group experienced fewer episodes of orthostatic hypotension than the tilt-table group, but that the tilt-table group improved consciousness levels more than the Erigo group, raises some important questions about the mechanism by which head-up tilt might work on changes in consciousness. It raises the possibility that short episodes of orthostatic hypotension have wider effects than simply on the cardiovascular system, and which may be stimulatory to the person in disordered consciousness resulting in improvements in consciousness. However, the finding that a tilt-table programme is more effective in changing levels of consciousness than the Erigo needs to be replicated in future studies before it can be trusted.^24^

Many physiological mechanisms might contribute to changes in consciousness or ‘wakefulness’, but they have not been fully investigated in this population. Certain physiological stimuli could increase levels of consciousness, including an increase in heart rate to maintain blood pressure when a person is elevated into a head-up position. Riberholt et al.^21^ have demonstrated that head-up tilt increases the heart rate of those in disorders of consciousness. An intact vestibular system will signal changes in motion to the brain even if the brain is in a disordered state of consciousness, so a change of position might stimulate increased alertness. Positioning a person in a head-up tilt can induce some noxious stimuli through stretching tight or spastic muscles, and discomfort might stimulate brain activity. Persons in disorders of consciousness can feel painful stimuli, in the sense that reliable cortical responses to painful laser stimulation have been detected in persons in both a vegetative state and minimally conscious state.^31^ The possibility that the physiological effects induced by head-up tilt might indirectly affect consciousness should be considered in future research.

The included studies grouped together diverse individuals with disorders of consciousness from different causation. Pronounced changes in a few individuals may have distorted the group average for change in consciousness. Well-designed single-case methodology with strict continuous measurement protocols and periods of withdrawing treatment may be a good way of investigating the effects of head-up tilt in more detail. Valid outcome measures, such as the Coma Recovery Scale-Revised that have been shown to differentiate different states of consciousness, are required. The full reporting of each participant’s characteristics and concurrent treatments in this type of study, for example their age, type of head injury, area of brain lesion and details of other therapies, would provide better transparency for clinicians. Overall, more research is required in this area to ascertain the effect of head-up tilt on consciousness in a prolonged disorders of consciousness population.

**Clinical messages**

- There is, as yet, insufficient evidence to require the use of the head-up tilt to raise consciousness in a prolonged disorders of consciousness population.
- Head-up tilt using an Erigo reduces the occurrence of orthostatic hypotension in a prolonged disorder of consciousness population.

## Supplemental Material

Supplemental_data – Supplemental material for A systematic review of head-up tilt to improve consciousness in people with a prolonged disorder of consciousness Click here for additional data file. Supplemental material, Supplemental_data for A systematic review of head-up tilt to improve consciousness in people with a prolonged disorder of consciousness by Harriet Ng and Andrew King in Clinical Rehabilitation
