# Supplementary material for: A systematic review of head-up tilt to improve consciousness in people with a prolonged disorder of consciousness
Source: Clin Rehabil. 2020 Jul 30;35(1):13–25. doi: 10.1177/0269215520946696 (PMC7814097; doi:10.1177/0269215520946696)
Supplement: Supplemental_data – Supplemental material for A systematic review of head-up tilt to improve consciousness in people with a prolonged disorder of consciousness [file Supplemental_data.pdf]

Table S1: Downs and Black Quality Assessment

	Checklist																											
	Reporting									External Validity			Internal Validity-Bias								Internal Validity-Confounders						Power	
	1	2	3	4	5	6	7	8	9	10	11	12	13	14	15	16	17	18	19	20	21	22	23	24	25	26	27	
First Author	Study aim	Main outcome	Subject characteristics	Description of Intervention	Principal confounders	Outcome data	Range of results	Adverse events	Lost to follow up	Probability value	Source population	Representative of population	Staff, place, facility	Subjects blind to intervention	Blind assessors	Data dredging	Same length of follow-up	Appropriate Statistical tests	Compliance with the	Accurate outcome measure	Control recruited same	Recruitment at same time	Randomised allocation	Concealed randomisation	Adjustment for confounders	Subjects lost to follow-up	0-5	TOTAL
Bartolo et al.[21]	1	1	1	0	2	1	1	1	1	1	1	0	1	0	0	0	0	1	1	0	0	1	0	0	0	1	0	16
Elliott et al.[3]	1	1	0	1	0	1	0	0	1	1	0	0	0	0	0	1	0	0	1	1	0	0	0	0	0	0	0	9
Frazzitta et al.[5]	1	1	1	1	2	1	1	1	1	1	1	1	1	0	1	1	0	1	1	1	1	1	1	0	0	1	0	23
Greco et al.[22]	1	1	1	1	0	1	1	0	1	1	0	0	0	0	0	0	1	1	1	0	0	0	0	0	0	0	0	11
Krewer et al.[6]	1	1	1	1	2	1	1	1	1	1	1	1	1	0	1	1	1	1	1	1	1	1	1	1	0	1	5	30
Luther et al.[10]	1	1	1	1	1	0	0	1	1	0	1	0	1	0	0	0	1	0	1	1	1	0	1	0	0	1	4	19
Riberholt et al.[24]	1	1	1	1	1	0	0	1	1	1	1	1	1	0	0	1	1	1	0	0	1	0	0	0	0	0	0	15
Taveggia et al.[23]	1	1	1	1	1	0	0	1	1	0	0	0	0	0	0	0	1	0	1	1	0	0	1	1	0	1	0	13
Toccolini et al.[25]	1	1	1	1	1	1	1	0	1	0	1	0	0	0	0	0	0	1	1	0	0	0	0	0	0	1	0	12
Wilson et al.[4]	1	1	1	1	1	1	0	0	1	0	0	0	1	0	0	1	1	0	1	1	0	0	0	0	0	0	0	12

Table S2: Secondary classification of Downs and Black checklist

Study category	Number of studies	Name of studies
Excellent	1	Krewer et al.[6]
Good	1	Frazzitta et al.[5]
Fair	3	Luther et al.[10], Riberholt et al.[24] , Bartolo et al.[21]
Poor	5	Elliott et al.[3], Greco et al.[22], Wilson et al.[4], Toccolini et al.[25], Taveggia et al.[23]

Appendix 1
Search Strategy 21/6/2020
CINAHL

MY EBSCOhost

Sunday, June 21, 2020 10:15:5

S34	S27 AND S30 AND S31	674
S33	S27 AND S29 AND S31	996
S32	S27 AND S28 AND S31	14
S31	S24 OR S25 OR S26	34,747
S30	S22 OR S23	151,70
S29	S18 OR S19 OR S20 OR S21	393,18
S28	S17	924

S27	S1 OR S2 OR S3 OR S4 OR S5 OR S6 OR S7 OR S8 OR S9 OR S10 OR S11 OR S12 OR S13 OR S14 OR S15 OR S16	523,44[illegible]
S26	(TI "Quantitative Electroencephalography") OR (AB "Quantitative Electroencephalography") OR (TI "Quantitative EEG") OR (AB "Quantitative EEG") OR (TI "Electroencephalography") OR (AB "Electroencephalography") OR (TI "EEG") OR (AB ""EEG")	9,761
S25	(TI "Level of consciousness") OR (AB "Level of consciousness") OR (TI "Consciousness Disorders") OR (AB "Consciousness Disorders") (TI "Arousal") OR (AB "Arousal") OR (TI "Wakefulness") OR (AB "Wakefulness") OR (TI "Alertness") OR (AB "Alertness")	8,387
S24	(TI "Coma Recovery Scale Revised") OR (AB "Coma Recovery Scale Revised") OR (TI "CRS-R") OR (AB "CRS-R") OR (TI "Glasgow coma scale") OR (AB "Glasgow coma scale") OR (TI "GCS") OR (AB "GCS") OR (TI "Wessex Head Injury Matrix") OR (AB "Wessex Head Injury Matrix") OR (TI "WHIM") OR (AB "WHIM") OR (TI "Sensory Modality Assessment and Rehabilitation Technique") OR (AB "Sensory Modality Assessment and Rehabilitation Technique") OR (TI "SMART") OR (AB "SMART") OR (TI "Sensory Tool to assess responsiveness") OR (AB "Sensory Tool to assess responsiveness") OR (TI "STAR") OR (AB "STAR") OR (TI "Neurobehavioral tool") OR (AB "Neurobehavioral tool")	17,275
S23	(MH "Physiotherapy") OR (TI "Physiotherapy") OR (AB "Physiotherapy") OR (MH "Physical Therapy") OR (TI "Physical Therapy") OR (AB "Physical Therapy") OR (AB "The Chartered Society of Physiotherapy") OR (AB "Australian Physiotherapy Association") OR (AB "Students, Physical Therapy") OR (TI "Physical Therapy Practice, Research-Based") OR (AB "Physical Therapy Practice, Research-Based") OR (AB "Physiotherapy Evidence Database) OR (AB "Canadian Physiotherapy Association") OR (AB "Physical Therapy Practice, Evidence-Based") OR (AB "Physical Therapy") OR (AB "Students, Physical Therapy")	55,553
S22	(MH "Rehabilitation") OR (TI "Rehabilitation") OR (AB "Rehabilitation") OR (TI "Rehab") OR (AB "Rehab)	105,51[illegible]
S21	(TI "Assisted stand") OR (AB "Assisted stand")	3
S20	(MH "Rise*") OR (TI "Rise*") OR (AB "Rise*")	37,970
S19	(MH "Stand*") OR (TI "Stand*") OR (AB "Stand*")	358,18[illegible]
S18	(TI "sit to stand") OR (AB "sit to stand")	1,680
S17	(TI "Tilt table") OR (AB "Tilt table") OR (TI "ERIGO") OR (AB "ERIGO") OR (AB "stand aid") OR (TI "stand aid") OR (TI "head up tilt") OR (AB "head up tilt")	924
S16	(MH "Minimally conscious state*") OR (AB "Minimally conscious state*") OR (TI "Minimally conscious state*") OR (TI "emergent minimally conscious state") OR (TI "MCS") OR (AB "MCS") OR (TI "emergent minimally conscious state") OR (AB "emergent minimally conscious state") OR (TI "EMCS") OR (AB "EMCS")	2,956
S15	(TI "Vegetative state*") OR (AB "Vegetative state*") OR (TI "VS") OR (AB "VS") OR (TI "Persistent Vegetative State") OR (AB "Persistent Vegetative State") OR (TI "'unresponsive wakefulness syndrome") OR (AB 'unresponsive wakefulness syndrome")	194,33[illegible]
S14	(MH "Carotid Sinus Syndrome") OR (AB "Carotid Sinus Syndrome") OR (TI "Carotid Sinus Syndrome") OR (MH "Syncope, Vasovagal") (AB "Syncope, Vasovagal") OR (TI "Syncope, Vasovagal")	114
S13	(MH "Coma") OR (MM "Coma") OR (AB "Coma") OR (TI "Coma") OR (MH "Unconsciousness") OR (AB "Unconsciousness") OR (TI "Unconsciousness")	10,660
S12	(MH "Consciousness Disorders") OR (TI "prolonged disorder of consciousness") OR (AB "prolonged disorder of consciousness") OR (TI "chronic disorder of consciousness") OR (AB "chronic disorder of consciousness") OR (TI "low awareness state") OR (AB "low awareness state")	938
S11	(MM "Neurobehavioral Manifestations+")	77,804
S10	(MH "Severe hypoglycaemia") OR (TI "Severe hypoglycaemia") OR (AB "Severe hypoglycaemia")	440
S9	(MH "Alcoholic Intoxication") OR (AB "Alcoholic Intoxication") OR (AB "Alcoholic Intoxication")	3,517
S8	(MH "Overdose") OR (TI "Overdose") OR (AB "Overdose") OR (MH "Street Drugs") OR (TI "Street Drugs") OR (AB "Street Drugs")	10,911

S7	(MH “Vasculitis, Central Nervous System”) OR (TI “Vasculitis, Central Nervous System”) OR (AB “Vasculitis, Central Nervous System”) OR (MH “Brain Abscess”) OR (TI “Brain Abscess”) OR (AB “Brain Abscess”) OR (MH “Meningitis, Bacterial”) OR (TI “Meningitis, Bacterial”) OR (AB “Meningitis, Bacterial”) OR (MH “Meningitis, Viral”) OR (TI “Meningitis, Viral”) OR (AB “Meningitis, Viral”) OR (MH “Encephalitis”) OR (TI “Encephalitis”) OR (AB “Encephalitis”)	9,655
S6	(MH “Hypoxia, Brain”) OR (TI “Hypoxia, Brain”) OR (AB “Hypoxia, Brain”) OR (TI “Hypoxia-Ischemia, Brain”) OR (AB “Hypoxia-Ischemia, Brain”) OR (TI “Altered Cerebral Tissue Perfusion (‘NANDA’)”) OR (AB “Altered Cerebral Tissue Perfusion (‘NANDA’)”) OR (MH “Cerebral Ischemia”) (TI “Cerebral Ischemia”) OR (AB “Cerebral Ischemia”) OR (TI “hypoxic brain injury”) OR (AB “hypoxic brain injury”)	4,145
S5	(MH “Brain injur*”) OR (TI "Brain injur*") OR (AB “"Brain injur*") (MH “Brain Damage, Chronic”) OR (TI “Brain Damage, Chronic”) OR (AB “Brain Damage, Chronic”) OR (MH “Brain Concussion”) OR (TI “Brain Concussion”) OR (AB “Brain Concussion”) OR (MH “Brain Contusions”) OR (TI “Brain Contusions”) OR (AB “skull fracture”)	29,409
S4	(MH “Cardiovascular Disease”) OR (TI “Cardiovascular Disease”) OR (AB “Cardiovascular Disease”) OR (MH “Cerebrovascular Disorders”) OR (TI “Cerebrovascular Disorders”) OR (AB “Cerebrovascular Disorders”) OR (AB “Aneurysm”) OR (AB “Arterial Occlusive Diseases”) OR (MH “Cerebral Arterial Diseases”) OR (TI “Cerebral Arterial Diseases”) OR (AB “Cerebral Arterial Diseases”) OR (MH “Arterial Occlusive Diseases”) OR (TI “Arterial Occlusive Diseases”) OR (AB “Arterial Occlusive Diseases”) OR (TI “Vasculitis, Central Nervous System”) OR (AB “Vasculitis, Central Nervous System”) OR (TI “Vasculitis, Central Nervous System”) OR (AB “Vasculitis, Central Nervous System”) OR (MH “Vertebral Artery Dissections”) OR (TI “Vertebral Artery Dissections”) OR (AB “Vertebral Artery Dissections”)	64,332
S3	(MH “Stroke”) OR (TI “Stroke”) OR (AB “Stroke”) OR (MH “Stroke, Lacunar”) OR (TI “Stroke, Lacunar”) OR (AB “Stroke, Lacunar”) OR (MH “Stroke Patients”) OR (TI “Stroke Patients”) OR (AB “Stroke Patients”) OR (TI “cerebral vascular accident”) OR (AB “cerebral vascular accident”)	114,59[illegible]
S2	(MM “Cerebrovascular Disorders+”) OR (MH “Basal Ganglia Cerebrovascular Disease”) OR (TI “Basal Ganglia Cerebrovascular Disease”) OR (AB “Carotid Artery Diseases”) OR (MH “Intracranial Embolism”) OR (TI “Intracranial Embolism”) OR (AB “Intracranial Embolism”) OR (MH “Thrombosis”) OR (TI “Thrombosis”) OR (AB “Thrombosis”) OR (MH “Intracranial Hemorrhage”) OR (TI “Intracranial Hemorrhage”) OR (AB “Intracranial Hemorrhage”) OR (MH “Intracranial Embolism and Thrombosis) OR (TI “Intracranial Embolism and Thrombosis”) OR (AB “Intracranial Embolism and Thrombosis) OR (MH “Carotid Artery Thrombosis”) OR (TI “Carotid Artery Thrombosis”) OR (AB “Carotid Artery Thrombosis”) OR (MH “Intracranial Embolism”) OR (TI “Intracranial Embolism”) OR (AB “Intracranial Embolism”) OR (MH “Carotid Artery Diseases”) OR (TI “Carotid Artery Diseases”) OR (AB “Carotid Artery Diseases”) OR (MH “Carotid Artery Dissections”) OR (TI “Carotid Artery Dissections”) OR (AB “Carotid Artery Dissections”) OR (MH “Carotid Artery Thrombosis”) OR (TI “Carotid Artery Thrombosis”) OR (AB “Carotid Artery Thrombosis”) OR (MH “Carotid Stenosis”) OR (TI “Carotid Stenosis”) OR (AB “Carotid Stenosis”) OR (MH “Moyamoya Disease”) OR (TI “Moyamoya Disease”) OR (AB “Moyamoya Disease”) OR (MH “Cerebral Hemorrhage”) OR (TI “Moyamoya Disease”) OR (AB “Moyamoya Disease”)	49,425
S1	(MH Brain Diseases) OR (MH “Central Nervous System Diseases”) OR (TI “Central Nervous System Diseases”) OR (AB “Central Nervous System Diseases”) OR (MH “Brain Diseases”) OR (TI ““Brain Diseases”) OR (AB “Brain Diseases”)	9,702

Search strategy
Medline 21/6/2020

MY EBSCOhost	Sunday, June 14, 2020 11:39:46 AM	
S216	S207 AND S200 AND S212	912
S215	S207 AND S209 AND S212	92
S214	S207 AND S187 AND S211	36
S213	(TI "stand aid") OR (AB "stand aid") OR (TI "Oswestry standing frame") OR (AB "Oswestry standing frame") OR (TI "standing frame") OR (AB "standing frame") OR (MH “Rise*”) OR (TI “Rise*”) OR (AB “Rise*”) OR (MH “Posture”) OR (TI “Posture”) OR (AB “Posture”)	346,765
S212	S203 OR S204 OR S205 OR S206	63,846
S211	S200 OR S201 OR S210	445,641
S210	(MH “Occupational Therapy”) OR (TI ““Occupational Therapy”) OR (AB “Occupational Therapy”) OR (MH “Occupational Therapy Department, Hospital”) OR (TI “Occupational Therapy Department, Hospital”) OR (AB “Occupational Therapy Department, Hospital”)	13,162
S209	S189 OR S190 OR S192 OR S193 OR S194 OR S195 OR S196 OR S197 OR S198 OR S199	348,778
S208	S187	1,333)
S207	S179 OR S181 OR S182 OR S183 OR S184 OR S185 OR S186	421,141
S206	(TI “Coma Recovery Scale Revised”) OR (AB “Coma Recovery Scale Revised”) OR (TI “CRS-R”) OR (AB “CRS-R”) OR (TI “Glasgow coma scale”) OR (AB “Glasgow coma scale”) (TI “GCS”) OR (AB “GCS”) OR (TI “Wessex Head Injury Matrix”) OR (AB	53,951

	"Wessex Head Injury Matrix") OR (TI "WHIM") OR (AB "WHIM") OR (TI "Sensory Modality Assessment and Rehabilitation Technique") OR (AB "Sensory Modality Assessment and Rehabilitation Technique") OR (TI "SMART") OR (AB "SMART") OR (TI "Sensory Tool to assess responsive ...	
S205	(MH "Alertness") OR (TI "Alertness") OR (AB "Alertness")	6,406
S204	(MH "Consciousness disorder") OR (TI "Consciousness disorder") OR (AB "Consciousness disorder")	155
S203	(MH "Level of consciousness") OR (TI "Level of consciousness") OR (AB "Level of consciousness")	3,691
S202	(TI "Tilt table") OR (AB "Tilt table") OR (TI "ERIGO") OR (AB "ERIGO")	1,333
S201	(MH "Physiotherapy") OR (TI "Physiotherapy") OR (AB "Physiotherapy") OR (TI "Physical Therapy Modalities") OR (AB "Physical Therapy Modalities") OR (TI "Physical Therapy Specialty") OR (AB "Physical Therapy Specialty")	18,384
S200	("Rehabilitation"+) OR (TI "Stroke Rehabilitation") OR (AB "Stroke Rehabilitation") OR (TI "Rehabilitation Research") OR (AB "Rehabilitation Research") OR (TI "Physical and Rehabilitation Medicine") OR (AB "Physical and Rehabilitation Medicine") OR (TI "Neurological Rehabilitation") OR (AB "Neurological Rehabilitation") OR (TI "Rehabilitation Nursing") OR (AB "Rehabilitation Nursing") OR (TI "Rehabilitation Centers") OR (AB "Rehabilitation Centers")	427,409
S199	(MH "Posture") OR (TI "Posture") OR (AB "Posture")	79,228
S198	(MH "Rise*") OR (TI "Rise*") OR (AB "Rise*")	268,623
S197	(AB "standing frame")	60
S196	(TI "standing frame")	17
S195	(TI "sitting balance") OR (AB "sitting balance")	300
S194	(TI "sit to stand") OR (AB "sit to stand")	2,347
S193	(TI "standing frame") OR (AB "standing frame")	66
S192	(AB "Oswestry standing frame")	3
S191	(MH "Coma") OR (TI "Coma") OR (AB "Coma") OR (MH "Coma, Post-Head Injury") OR (TI "Coma, Post-Head Injury") OR (AB "Coma, Post-Head Injury") OR (MH "Vegetative state") OR (TI "Vegetative state") OR (AB "Vegetative state") OR (TI "VS") OR (AB "VS") OR (MH "Persistent Vegetative State") OR (TI "Persistent Vegetative State") OR (AB "Persistent Vegetative State") OR (TI "'unresponsive wakefulness syndrome") OR (AB 'unresponsive wakefulness syndrome") OR (MH "Minimally conscious state*") OR (TI "Mi ...	776,499
S190	(TI "Oswestry standing frame")	1
S189	(MH "stand aid") OR (TI "stand aid") OR (AB "stand aid")	0
S188	(MH "stand aid") OR (TI "stand aid") OR (AB "stand aid")	0
S187	(MH "Tilt table") OR (TI "Tilt table") OR (AB "Tilt table") OR (MH "ERIGO") OR (TI "ERIGO") OR (AB "ERIGO")	1,333
S186	(MH "Vegetative state") OR (TI "Vegetative state") OR (AB "Vegetative state") OR (MH "Persistent Vegetative State") OR (TI "Persistent Vegetative State") OR (AB "Persistent Vegetative State") OR (MH "Coma, Post-Head Injury") OR (TI "Coma, Post-Head Injury") OR (AB "Coma, Post-Head Injury") OR (MH "Minimally conscious state*") OR (TI "Minimally conscious state*") OR (AB "Minimally conscious state*")	5,006
S185	(MH "Consciousness Disorders") OR (TI "Consciousness Disorders") OR (AB "Consciousness Disorders") OR (TI "Consciousness") OR (AB "Consciousness")	36,276

S184	(MH “Encephalitis”) OR (TI “Encephalitis”) OR (AB “Encephalitis”) (TI “Anti-N-Methyl-D-Aspartate Receptor Encephalitis”) OR (AB “Anti-N-Methyl-D-Aspartate Receptor Encephalitis “Cerebral Ventriculitis”) OR (TI “Infectious Encephalitis”) OR (AB “Infectious Encephalitis”) OR (TI “Encephalitis, Viral”) OR (AB “Encephalitis, Viral”) (TI “Limbic Encephalitis”) OR (AB “Limbic Encephalitis”)	33,947
S183	(MH “Vasculitis”) OR (TI “Vasculitis”) OR (AB “Vasculitis”) OR (TI “Vasculitis, Central Nervous System”) OR (AB “Vasculitis, Central Nervous System”)	36,380
S182	(MH “Hypoxia, Brain”) OR (TI “Hypoxia, Brain”) OR (AB “Hypoxia, Brain”) OR (MH “Hypoxia-Ischemia, Brain”) OR (TI “Hypoxia-Ischemia, Brain”) OR (AB “Hypoxia-Ischemia, Brain”)	12,795
S181	(MH “Brain Injury, Chronic”) OR (TI “Brain Injury, Chronic”) OR (AB“Brain Injury, Chronic”) OR (MH “Brain Injuries”) OR (TI “Brain Injuries”) OR (AB “Brain Injuries”) OR (MH “Brain Injuries, Traumatic”) OR (TI “Brain Injuries, Traumatic”) OR (AB “Brain Injuries, Traumatic”) OR (MH “Brain Injuries, Diffuse”) OR (TI “Brain Injuries, Diffuse”) OR (AB “Brain Injuries, Diffuse”) OR (MH “Cerebrovascular Trauma”) OR (TI “Cerebrovascular Trauma”) OR (AB “Cerebrovascular Trauma”) OR (MH “Head Injuries, ...	69,495
S180	(TI “Severe hypoglycaemia”) OR (AB “Severe hypoglycaemia”)	1,238
S179	(MH “Stroke”) OR (AB “Stroke”) OR (TI Stroke”) OR (MH “Stroke, Lacunar”) OR (AB “Stroke, Lacunar”) OR (TI “Stroke, Lacunar”) OR (MH “Stroke Rehabilitation”) OR (AB “Stroke Rehabilitation”) OR (TI “Stroke Rehabilitation”) OR (TI “National Institute of Neurological Disorders and Stroke”) OR (AB “National Institute of Neurological Disorders and Stroke”)	240,484
